# Association Between Lifestyle Parameters, Quality of Life, and Satisfaction with Life in Chilean University Students

**DOI:** 10.3390/healthcare13161950

**Published:** 2025-08-09

**Authors:** Felipe Caamaño-Navarrete, Carlos Arriagada-Hernández, Gerardo Fuentes-Vilugrón, Iris Paola Guzmán-Guzmán, Lorena Jara-Tomckowiack, Daniel Jerez-Mayorga, Indya del-Cuerpo, Guido Contreras-Díaz, Claudio Hernández-Mosqueira, Claudia Andrea Vargas, Pedro Delgado-Floody

**Affiliations:** 1 Physical Education Career, Faculty of Education, Universidad Autónoma de Chile, Temuco 4780000, Chile; marfel77@gmail.com (F.C.-N.); carlos.arriagada@uautonoma.cl (C.A.-H.); 2 Collaborative Research Group for School Development (GICDE), Temuco 4780000, Chile; gerardo.fuentes@uautonoma.cl; 3 Faculty of Education, Universidad Autónoma de Chile, Temuco 4780000, Chile; 4 Faculty of Chemical-Biological Sciences, Universidad Autónoma de Guerrero, Chilpancingo 39000, Guerrero, Mexico; pao_nkiller@yahoo.com.mx; 5 Faculty of Education, Universidad Católica de Temuco, Temuco 4780000, Chile; lorenajarat@gmail.com; 6 Exercise and Rehabilitation Sciences Institute, School of Physical Therapy, Faculty of Rehabilitation Sciences, Universidad Andres Bello, Santiago 7591538, Chile; djerezmayorga@ugr.es; 7 Department of Physical Education and Sport, Faculty of Sports Sciences, University of Granada, 18012 Granada, Spain; delcuerpo@ugr.es; 8 Escuela de Kinesiología, Facultad de Ciencias de la Rehabilitación Y Calidad de Vida, Universidad San Sebastián, Lago Panguipulli 1390, Puerto Montt 5501842, Chile; guido.contreras@uss.cl; 9 Departamento de Ciencias de la Educación, Universidad del Bio-Bio, Chillán 3800708, Chile; chernandez@ubiobio.cl; 10 Department of Physical Education, Sport and Recreation, Universidad de la Frontera, Temuco 4811230, Chile; pedro.delgado@ufrontera.cl

**Keywords:** mental health, quality of life, lifestyle, physical activity, screen time, students

## Abstract

**Background:** A negative lifestyle has been reported to be associated with poor quality of life (QoL). However, there is limited information regarding the relationship between satisfaction with life (SWL) and lifestyle factors in university students. **Objective:** The aim of the present study was to determine the association between lifestyle parameters, quality of life (i.e., physical, psychological, social, and environmental dimensions), and categories of satisfaction with life (i.e., extremely satisfied, satisfied, slightly satisfied, dissatisfied, extremely dissatisfied) in Chilean university students. **Methods:** This cross-sectional study included a total of 212 university students (128 females and 83 males), aged between 18 and 28 years. Physical activity (PA), screen time (ST), dietary habits, sleep quality, QoL, and SWL were assessed using validated questionnaires. **Results:** SWL presented inverse association with unhealthy diet (β = −0.18, 95%CI; −0.28 to −0.09, *p* < 0.001), sleep < 6 h (β = −0.21, 95%CI; −0.31 to −0.11, <0.001), ST > 4 h (β = −0.10, 95%CI; −0.18 to −0.01, 0.015) and non-PA (β = −0.10, 95%CI; −0.19 to −0.01, *p* = 0.027). Regarding the association between lifestyle parameters and QoL, unhealthy diet was inversely associated with the physical (β; 0.18, 95%CI; −0.27 to −0.09, <0.001), psychological (β = −0.18, 95%CI; −0.27 to 0.09, *p* < 0.001), and environmental (β = −0.14, 95%CI; −0.23 to −0.06, 0.001) dimensions, and overall QoL score (β = −0.06, 95%CI; −0.09 to −0.03, *p* < 0.001). Sleep duration < 6 h showed inverse associations with the physical (β = −0.21, 95%Ci; −0.30 to −0.11, <0.001), psychological (β = −0.20, 95%CI; −0.30 to −0.10, <0.001), social (β = −0.30, 95%CI; −0.47 to −0.14, *p* < 0.001), and environmental (β = −0.13, 95CI; −0.21 to −0.04, *p* = 0.004) dimensions, and overall QoL score (β = −0.07, 95%CI; −0.10 to −0.04, *p* < 0.001). Non-PA was associated with physical dimensions (β = −0.09, 95%CI; −0.17 to −0.01, *p* = 0.019). Non-PA reported association with physical (β = −0.16, 95%CI; −0.25 to −0.07, *p* < 0.001), psychological (β = −0.20, 95%CI; −0.30 to −0.10, *p* < 0.001), social (β = −0.25, 95%CI; −0.41 to −0.09, *p* = 0.002), and environmental (β = −0.11, 95%CI; −0.19 to −0.02, *p* = 0.010) dimensions, and QoL overall score (β = −0.06, 95%CI; −0.09 to −0.03, *p* < 0.001). **Conclusions:** Poor diet, inadequate sleep, excessive ST, and non-PA are all associated with negative impacts on SWL and QoL in Chilean university students.

## 1. Introduction

Mental health has become an increasingly important topic among university students [1]. It is estimated that around one billion people worldwide suffer from a mental disorder [2]. The World Health Organization Quality of Life Group defines quality of life (QoL) as “individuals’ perception of their position in life in the context of the culture and value systems in which they live and in relation to their goals, expectations, standards and concerns” [3]. Moreover, QoL is considered a complex and multifaceted concept which encompasses various aspects of people’s lives [4]. In the university context, QoL and satisfaction with life (SWL) are closely related concepts, reflecting a balance between students’ external and internal resources [5]. In addition, several studies have indicated that QoL is influenced by multiple factors, including lifestyle habits [6].

SWL is defined as a widely used construct in well-being research [7] and is understood as the overall assessment individuals make about their own lives, based on their beliefs and behaviors. Previous evidence has shown that SWL reflects a cognitive and subjective evaluation of one’s life [7,8]. Furthermore, SWL represents a global self-assessment component of well-being, encompassing physical, mental, and social dimensions [9]. It is often recognized as a core element of psychological well-being [10], and it is considered an important predictor of both mental and physical health [11]. Research also suggests that SWL is influenced by a complex combination of immediate, intermediate, and long-term factors [12]. For example, university students often experience significant psychological pressure due to academic competition and related adjustment challenges [5].

Unhealthy lifestyle habits have become a topic of growing interest due to their consequences during the university period. Evidence suggests that the transition from home to university may negatively affect students’ lifestyle behaviors, including dietary habits, screen time (ST), physical activity (PA), and sleep patterns [13,14]. This stage of life has been linked to unhealthy lifestyle choices [15,16], which may impact various dimensions of mental health.

Scientific evidence increasingly supports the idea that lifestyle factors significantly influence mental health outcomes [17]. For instance, Caamaño-Navarrete et al. (2024) [18] reported strong associations between unhealthy lifestyles and poorer QoL in university students. Moreover, parameters such as diet, sleep, ST, and PA have been shown to affect mental health in this population [19]. A systematic review by Salomou et al. (2023) [13] found that healthier diets among university students were associated with better mental health, as measured by lower scores on depression, anxiety, and stress scales. Another study reported that healthy eating habits were linked to higher SWL levels in university students [20]. Conversely, mental health problems such as depression and anxiety have been negatively associated with unhealthy eating, increasing the risk of life dissatisfaction [21]. Life satisfaction is considered a fundamental component of psychological well-being, and several studies have shown that lower SWL levels are associated with higher levels of anxiety. For example, Tsitsas et al. (2019) [22] found that students with lower anxiety scores reported higher SWL compared to those with higher stress levels.

In addition, the study by Lemola et al. (2015) [23] demonstrated that prolonged ST is negatively associated with depressive symptoms and anxiety in adolescents and young adults. Likewise, sleep deprivation is linked to an increased risk of depressive symptoms and difficulties in emotional regulation [24], emphasizing the importance of promoting healthier sleep habits among university students. Physical activity is also a fundamental aspect of psychological well-being [25]. Evidence shows that regular exercise is positively associated with reduced depressive symptoms and improved mental health among university students.

This study contributes to the growing body of scientific evidence that supports the design of interventions aimed at improving QoL and SWL in university students. It specifically focuses on lifestyle parameters such as diet, sleep habits, screen time, and physical activity. The findings underscore the importance of addressing these lifestyle factors to enhance mental health and well-being in this population. Furthermore, a study by García-Pérez et al. (2025) [26] highlighted the importance of promoting healthy lifestyle behaviors to improve mental health outcomes in university students. A negative lifestyle has been shown to be associated with poorer mental health and QoL in Chilean university students.

The novelty of this study lies in its focus on the Chilean university student population, providing insights into the particular characteristics and needs of this group. Given the cross-sectional nature of the research, the study hypothesizes that an unhealthy lifestyle is negatively associated with both QoL and SWL. Our hypothesis is based on the theoretical model proposed by Decy and Ryan (2013), who argue that motivation is fundamental in promoting autonomous or controlled behaviors [27,28]. This theoretical model indicates that intrinsic motivation (the most self-determined; activity for one’s own pleasure), extrinsic motivation (activity for external recognition), and demotivation (the least self-determined) have a positive effect on quality of life and life satisfaction, and psychological well-being [29,30]. For example, it has been indicated that there is a strong tendency to improve physiological areas with an increase in healthy lifestyle choices [31]. In other words, quality of life and life satisfaction consider different aspects of human well-being, since the former considers bio–psycho–social elements and life satisfaction includes the subjective aspects of human beings, which are influenced by internal motivations and basic psychological needs.

In light of this background, the aim of the present study was to determine the associations between lifestyle parameters and QoL (i.e., physical, psychological, social, and environmental dimensions), as well as categories of satisfaction with life (i.e., extremely satisfied, satisfied, slightly satisfied, dissatisfied, extremely dissatisfied) among Chilean university students.

## 2. Materials and Methods

### 2.1. Participants

This was a cross-sectional study involving 211 university students (128 women and 83 men), aged between 18 and 28 years. A total of 43 participants (26 females and 17 males) were excluded due to not meeting the inclusion criteria or for other unspecified reasons. Sample size calculation was performed considering the following factors: (1) enrolment of students from the University (UA CHILE), (2) a significance level of 5%, (3) an absolute precision of 5%, (4) a statistical power of 95%, (5) the statistical test (Lineal Multiple Regression), and (6) an effect size of 0.15. Based on these parameters and accounting for an expected response rate of 80%, a sample size of 148 university students was used [32].

The inclusion criteria required participants to provide informed consent and to be enrolled as students in the Faculty of Education. The exclusion criteria included any medical contraindications that could interfere with participation in the evaluations, as well as absence during the assessment period.

The study complied with the principles outlined in the Declaration of Helsinki (2013) and was approved by the Ethics Committee of the Universidad Autónoma de Chile, Chile (Protocol No. CEC 18–23). All questionnaires were completed individually on the university campus in the presence of trained research assistants, who were available to address any questions or concerns. Data collection took place during the academic year, specifically in morning classes.

### 2.2. Main Outcomes

#### 2.2.1. Lifestyle

PA was assessed using the short form of the International Physical Activity Questionnaire (IPAQ-SF) [33]. The IPAQ-SF includes seven items addressing the frequency, duration, and intensity (moderate and vigorous) of PA during the previous seven days. This instrument has been previously validated in Chilean populations [34] and has been widely used among university students [35]. The IPAQ-SF has shown moderate internal consistency (Cronbach Alpha  =  0.65) [36].

ST and sleep duration were evaluated using the following questions: “How many hours a week do you watch videos?”, “How many hours a week do you play video games?”, and “How many hours do you usually sleep per day and/or night?”. These items have been employed in previous research [24,33].

Eating habits were assessed through a questionnaire previously used in studies involving Chilean university students [18]. The instrument includes 15 dichotomous (yes/no) items evaluating dietary habits, including the frequency of consumption of specific foods, adequacy of food intake, and behaviors related to healthy eating. Based on the total score, eating habits were categorized as follows: ≥13 points, healthy; 10–12 points, on the right track but could improve; 7–9 points, unhealthy; and ≤6 points, very unhealthy [37].

#### 2.2.2. Quality of Life

QoL was assessed using the WHOQOL-BREF instrument, an abbreviated version of the World Health Organization Quality of Life Questionnaire. This instrument comprises 26 items distributed across four key domains that reflect essential dimensions of human experience. Responses are recorded on a five-point Likert scale, allowing participants to express their perceptions based on their experiences during the two weeks preceding the assessment. The four domains evaluated by the WHOQOL-BREF are as follows: (a) Physical health, which includes aspects such as energy, fatigue, and pain; (b) Psychological health, which assesses mood, self-esteem, and cognitive functions; (c) Social relationships, which examine social support and personal interactions; and (d) Environment, which addresses satisfaction with the physical environment, safety, and access to resources. Higher scores on the WHOQOL-BREF indicate a better perceived quality of life. This instrument has been validated in various populations, including university students [38,39], and has demonstrated its utility in studies involving Chilean adults, supporting its applicability in the present research [40]. The WHOQOL-BREF has shown strong internal consistency with a reported Cronbach’s alpha of 0.88 [40].

#### 2.2.3. Life Satisfaction

Satisfaction with life was assessed using the Satisfaction With Life Scale (SWLS), which evaluates global cognitive judgments regarding individuals’ overall perception of their lives. The instrument consists of five items, including statements such as: “In most ways my life is close to my ideal”, “The conditions of my life are excellent”, “I am satisfied with my life”, “So far I have achieved the important things I want in life”, and “If I could live my life again, I wouldn’t change almost anything”. Responses were rated on a five-point Likert scale ranging from “strongly disagree” to “strongly agree”, with higher scores indicating greater satisfaction with life [41]. The SWLS has been validated and widely applied among university student populations, demonstrating its reliability and appropriateness for similar research contexts [20]. The scale has shown acceptable internal consistency, with a reported Cronbach’s alpha of 0.82 [42].

### 2.3. Statistical Analysis

Statistical analyses were conducted using STATA version 15.0. The Shapiro–Wilk test was used to assess the normality of the data distribution. Continuous variables are presented as medians and standard deviations, while categorical variables are expressed as proportions. Differences between categorical variables were assessed using the Chi-square test. To examine the relationship between QoL, SWL, and lifestyle parameters, logistic regression models were applied, adjusting for age and sex. The results are reported as beta coefficients (β; 95% confidence interval [CI]) and relative risk (RR). The beta coefficient (β) represents the estimated change in the outcome variable (lifestyle parameters) for a one-unit change in the predictor variable (QoL or SWL). Relative risk indicates the magnitude of the association between exposure variables and the outcome, providing an estimate of the likelihood of a given outcome occurring in relation to the exposure.

## 3. Results

Table 1 presents the descriptive characteristics of the study sample.

The baseline characteristics of the study population are summarized in Table 1. A total of 211 Chilean university students (128 women and 83 men), aged between 18 and 28 years, participated in the study. The median age of the participants was 21 years. Among the QoL domains, median scores were higher for the physical and environmental dimensions compared to the psychological and social domains. Lifestyle parameters were found to be within regular ranges. Regarding satisfaction with life, approximately 47% of participants reported being satisfied, whereas around 43% reported being dissatisfied.

**Figure 1:**
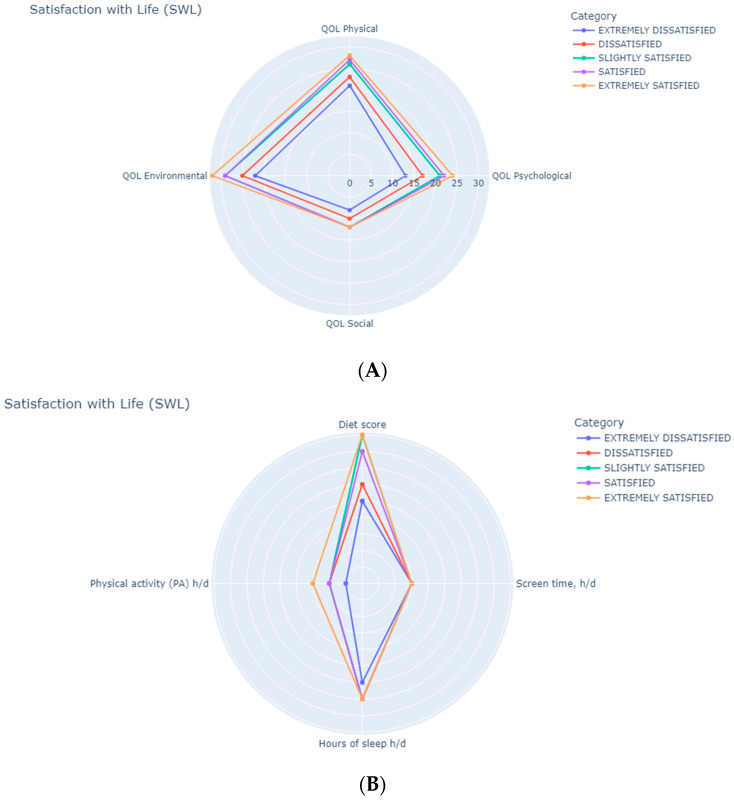
Radar chart showing median values of the association between satisfaction with life and QoL parameters (**A**), and lifestyle parameters (**B**).

Figure 1 shows the radar chart illustraing the median values of the different SWL categories across the various QoL dimensions. As shown in Figure 1A, the physical, psychological, environmental, and social domains were all positively associated with the “extremely satisfied” SWL category (all *p* < 0.001). In terms of lifestyle parameters (Figure 1B), participants in the “extremely satisfied” SWL category showed a positive association with healthier eating habits (*p* < 0.001) and greater sleep duration (hours per day) (*p* = 0.002).

Table 2 presents the RR associated with different levels of SWL in relation to poor lifestyle parameters. Participants classified as “extremely dissatisfied” with life were significantly more likely to exhibit unhealthy behaviors. Specifically, being extremely dissatisfied was associated with poor diet (RR = 5.2, 95% CI: 2.05–13.4, *p* = 0.001), poor sleep quality (RR = 3.74, 95% CI: 1.53–9.14, *p* = 0.004), and low levels of physical activity (RR = 3.87, 95% CI: 1.35–11.1, *p* = 0.012). These findings suggest that extremely low SWL is strongly associated with adverse lifestyle patterns.

Table 3 presents the associations between SWL and QoL with various lifestyle parameters. SWL showed a significant inverse association with poor diet (β = −0.18, 95% CI: −0.28 to −0.09, *p* < 0.001), sleep duration of less than 6 h (β = −0.21, 95% CI: −0.31 to −0.11, *p* < 0.001), ST greater than 4 h per day (β = −0.10, 95% CI: −0.18 to −0.01, *p* = 0.015), and non-PA (β = −0.10, 95% CI: −0.19 to −0.01, *p* = 0.027).

Regarding the associations between lifestyle parameters and QoL domains, poor diet was inversely associated with the physical (β = −0.18, 95% CI: −0.27 to −0.09, *p* < 0.001), psychological (β = −0.18, 95% CI: −0.27 to −0.09, *p* < 0.001), and environmental (β = −0.14, 95% CI: −0.23 to −0.06, *p* = 0.001) dimensions, as well as with the overall QoL score (β = −0.06, 95% CI: −0.09 to −0.03, *p* < 0.001).

Sleeping less than 6 h per day was inversely associated with the physical (β = −0.21, 95% CI: −0.30 to −0.11, *p* < 0.001), psychological (β = −0.20, 95% CI: −0.30 to −0.10, *p* < 0.001), social (β = −0.30, 95% CI: −0.47 to −0.14, *p* < 0.001), and environmental (β = −0.13, 95% CI: −0.21 to −0.04, *p* = 0.004) domains, as well as with the overall QoL score (β = −0.07, 95% CI: −0.10 to −0.04, *p* < 0.001).

Screen time ≥ 4 h per day was significantly associated with lower scores in the physical dimension (β = −0.09, 95% CI: −0.17 to −0.01, *p* = 0.019).

Physical inactivity was inversely associated with all QoL domains: physical (β = −0.16, 95% CI: −0.25 to −0.07, *p* < 0.001), psychological (β = −0.20, 95% CI: −0.30 to −0.10, *p* < 0.001), social (β = −0.25, 95% CI: −0.41 to −0.09, *p* = 0.002), and environmental (β = −0.11, 95% CI: −0.19 to −0.02, *p* = 0.010), in addition to the overall QoL score (β = −0.06, 95% CI: −0.09 to −0.03, *p* < 0.001).

## 4. Discussion

The objective of the present study was to determine the association between lifestyle parameters and QoL, including physical, psychological, social, and environmental domains, as well as SWL categories (i.e., extremely satisfied, satisfied, slightly satisfied, dissatisfied, extremely dissatisfied) in Chilean university students.

This study provides insights into the complex relationships between lifestyle behaviors, QoL, and SWL in this population, revealing significant associations that underscore the importance of promoting healthy habits among university students. Regarding QoL, poor diet was inversely associated with physical, psychological, environmental domains, and overall QoL scores. These findings align with previous research emphasizing the fundamental role of nutrition in physical and mental well-being [43]. Furthermore, students who reported higher levels of SWL also demonstrated healthier eating behaviors, including regular breakfast and dinner consumption, higher intake of dairy products, fruits, and vegetables, and a greater awareness of the importance of food for well-being [20]. These results are consistent with recent literature highlighting the influence of dietary patterns on overall well-being in young adults [21].

In line with this, prior studies have also reported a positive association between healthy eating and SWL in university students [44]. For example, research has found that both healthy diets and family mealtime interactions were positively related to SWL [45]. One study indicated that students with higher SWL were more likely to have regular eating patterns, such as eating breakfast (66.7%) and dinner (37.9%) daily at home, and consuming milk/dairy (54.5%), fruits (41.7%), and vegetables (57.6%) on a daily basis, while also perceiving food as important for their overall well-being [20]. These findings suggest that healthy lifestyle factors, such as dietary habits, should be targeted in preventive health interventions.

In the present study, poor diet was negatively associated with physical, psychological, and environmental QoL dimensions. These results are supported by previous research indicating that unhealthy lifestyle behaviors are significantly linked to poorer mental health and QoL in Chilean university students [18]. Similarly, López-Olivares et al. (2023) [46] found that adults adhering to healthy dietary patterns reported higher vitality, improved social functioning, and better mental health-related QoL. The Mediterranean diet, in particular, has been identified as a contributor to enhanced QoL. Further evidence also supports the association between healthy eating and improved QoL in both students and academic staff [47], with several studies indicating that adherence to nutritious dietary patterns positively affects overall QoL [4,48]. Consequently, efforts to improve dietary quality during the university years may reduce mental health problems and enhance students’ well-being [13].

Regarding sleep quality, SWL was found to be negatively associated with poor sleep. Students who reported sleeping less than six hours per day exhibited lower scores in physical, psychological, social, and environmental QoL domains, along with reduced overall QoL scores. Similarly, prolonged screen time (≥4 h/day) was associated with lower QoL scores. These results align with recent research showing that sleep deprivation and excessive screen time are significant predictors of poorer mental and physical health in university students [23,49,50,51]. The stress of academic demands, combined with widespread digital distractions, likely exacerbates these effects. In this regard, Bækø Ness and Saksvik-Lehouillier et al. (2018) [52] found that better sleep quality and longer sleep duration were positively associated with SWL in university students. Likewise, Pilcher et al. (1997) [53] reported that sleep quality was linked to both general health and life satisfaction. Rezaei et al. (2020) [54] similarly observed that sleep disturbances were negatively associated with QoL in this population. Furthermore, the study by Lavados-Romo et al. (2023) [55] found that university students with higher screen time exposure showed lower QoL, particularly in social and psychological domains. Screen use, especially in excess, may adversely affect emotional regulation and mental well-being in young adults [56].

In addition, our findings showed that low levels of PA were associated with lower SWL and QoL scores. The lack of PA was inversely related to all QoL dimensions, highlighting the essential role of regular exercise in maintaining both physical and mental health. While the statistical associations were significant, it is particularly important to note the consistency of the inverse relationship across all QoL domains. These findings are in line with existing literature demonstrating the positive effects of physical activity on QoL and SWL in young adults [25,57,58]. The benefits of regular physical exercise include reduced stress, enhanced mood, and improved overall health status [59,60,61,62]. Moreover, physical activity has been shown to improve SWL among university students [63], and it has been positively associated with self-efficacy, resilience, and psychological well-being [64]. Given this evidence, the promotion of physical activity should be considered a key component of interventions aimed at improving mental health and well-being in university populations [63].

Mental health and its components are a dimension of growing interest for universities [65]. Over the past few years, colleges have dealt with students who report severe mental health issues, so they need to be prepared to deal with this need [66]. Particularly in Chile, the First National University Mental Health Survey reported that 46% of university students had symptoms of anxiety, in addition to 54% indicated problems associated with stress. Another study conducted among Chilean university students showed a high prevalence of mental health problems [67]. On the other hand, a meta-analysis highlighted the importance of having a healthy lifestyle (which includes physical activity) due to the benefits it provides at the mental health level [68], which is relevant for universities that want to generate strategies to improve the mental health of their students. Although physical activity interventions within theoretical classes have gained popularity among teachers in schools [69]—a practice which can be transformed into an opportunity at the university level [70]—there is little evidence of this type of intervention in Chile or in the context of higher education. In addition, the popularity of such interventions has been reported in university students, who spend most of their classes in seated activities [71]. Likewise, Ferrer and Laughlin suggest that using active breaks in university classes could be a reasonable intervention methodology, since this does not require work outside of the classroom. In addition, such interventions can generate an entertaining and positive environment, improving levels of physical activity among this population [71]. Following this line of investigation, an intervention to incorporate active breaks of 5 to 10 min at the beginning of theoretical classes was shown to improve mental health variables and health-related quality of life, thus presenting a promising strategy to improve different parameters in higher education students [72]. The above, together with dietary recommendations, could be beneficial strategies to develop in the university context.

### Implications and Future Directions

The findings of this study have important implications for the design and implementation of targeted interventions aimed at promoting healthy lifestyles among Chilean university students. Educational programs that emphasize the importance of balanced nutrition, sleep hygiene, and regular physical activity are essential. In addition, universities should consider adopting institutional policies that support and facilitate healthy behaviors. These may include ensuring access to affordable and nutritious food options on campus, creating environments that encourage physical activity (e.g., sports facilities, active breaks), and increasing awareness of the importance of adequate sleep for both academic performance and overall well-being.

Several limitations should be acknowledged. First, the cross-sectional design of this study precludes the establishment of causal relationships between lifestyle parameters, QoL, and SWL. Longitudinal studies are needed to explore the temporal and potentially bidirectional relationships among these variables. Second, the study relied on self-reported data, which may be subject to recall and social desirability biases. Future research should consider incorporating objective measures—such as accelerometers for physical activity and actigraphy or validated sleep-tracking devices—to enhance the accuracy of behavioral assessments. In addition, another limitation was sample selection bias, since only students from one institution were considered, which could affect generalizability to other populations. Regarding the findings, this study reveals associations without delving into the causality of the variables (quality of life and life satisfaction). Moreover, future studies should examine how lifestyle changes over time impact QoL and SWL in university students, and evaluate the effectiveness of targeted interventions aimed at improving these outcomes. Another limitation was not considering other variables that could affect the model, such as socioeconomic status, study load, or urban/rural context, which could influence results.

## 5. Conclusions

In conclusion, this study highlights the critical role of lifestyle factors, particularly dietary habits, sleep quality, and physical activity, in shaping QoL and SWL among Chilean university students. These findings underscore the need for Chilean universities to monitor and improve students’ lifestyle behaviors through specific health promotion strategies. This is because the university stage is considered a period marked by important physical, emotional, and social transitions. In this sense, there is a constant challenge to recognize that healthy lifestyle habits not only contribute to the individual development of participants but also positively impact other areas of human development, such as academic performance and mental health, which are concerns at this stage. Furthermore, by promoting balanced nutrition, adequate sleep, and regular physical activity, institutions can contribute significantly to the overall well-being of this vulnerable population. Similarly, the implementation of multidisciplinary institutional strategies is strongly recommended, including educational programs and support policies that promote healthy environments on campus. However, institutional strategies must be comprehensive and contextualized to the social and cultural reality. Finally, future research should focus on evaluating the effectiveness of these interventions through longitudinal and experimental designs, particularly within the Latin American context, where evidence remains limited but highly needed.

## Figures and Tables

**Table 1:** Descriptive characteristics of lifestyle parameters and quality of life.

Demographic Characteristics	n = 211
Age (years) ^a^	21.6 ± 3.14
Sex, *n* (%) ^b^	
Women	128 (60.66)
Men	83 (39.34)
QoL parameters (score)	
QoL physical	25.2 ± 4.82
QoL psychological	19.5 ± 4.84
QoL social	10.9 ± 2.71
QoL environmental	27.7 ± 4.91
QoL raw score	83.3 ± 14.44
Lifestyle parameters	
Diet score ^a^	7.2 ± 3.07
Sleep time ^a^	6.8 ± 1.43
Screen time ^a^	2.7 ± 0.82
Physical activity time ^a^	2.1 ± 1.93
Satisfaction with life, score ^a^	15.5 ± 4.7
SWL category, *n* (%) ^b^	
Extremely satisfied	35 (16.6)
Satisfied	65 (30.8)
Slightly satisfied	19 (9.0)
Dissatisfied	72 (34.1)
Extremely dissatisfied	20 (9.5)

Data are expressed as median and standard deviation ^a^, and as proportions ^b^. QoL = Quality of Life; QoL parameters refer to the physical, psychological, social, and environmental dimensions.

**Table 2:** Comparison of the proportions of lifestyle categories according to levels of life satisfaction, and association between the presence of negative lifestyle categories and levels of dissatisfaction.

Parameter Lifestyle	DIET			*p* Value	RR * (CI95%), *p* Value
	GOOD	REGULAR	BAD		
SWL Category, *n* (%) ^b^				<0.001	
Extremely satisfied	12 (34.3)	16 (45.7)	7 (20.0)		1.0
Satisfied	20 (30.77)	33 (50.8)	12 (18.46)		1.05 (0.58–1.90),0.86
Slightly satisfied	7 (36.84)	8 (42.1)	4 (21.05)		0.97 (0.43–2.17), 0.94
Dissatisfied	9 (12.5)	33 (45.83)	30 (41.67)		2.29 (1.25–4.18), 0.007
Extremely dissatisfied	1(5.0)	6 (30.0)	13 (65.0)		5.2 (2.05–13.4), 0.001
**Parameter lifestyle**	**SLEEP QUALITY**				
	GOOD (≥8 h)	REGULAR (6–7 h)	BAD (<7 h)		
SWL Category, *n* (%) ^b^				0.006	
Extremely satisfied	14 (40.0)	18 (51.43)	3 (8.57)		1.0
Satisfied	26 (40.0)	33 (50.77)	6 (9.23)		1.0 (0.52–1.95), 0.97
Slightly satisfied	5 (26.32)	13 (68.42)	1 (5.26)		1.29 (0.53–3.1), 0.56
Dissatisfied	16 (22.22)	38 (52.78)	18 (25.0)		2.4 (1.22–4.5), 0.010
Extremely dissatisfied	3 (15.0)	9 (45.0)	8 (40.0)		3.74 (1.53–9.14), 0.004
**Parameter lifestyle**	**SCREEN TIME**				
	RECOMENDABLE (≤2 h)	REGULAR (3 h)	BAD (≥4 h)		
SWL Category, *n* (%) ^b^				0.542	
Extremely satisfied	20 (57.14)	11 (31.43)	4 (11.42)		1.0
Satisfied	33 (50.77)	20 (30.77)	12 (18.46)		1.32 (0.75–2.34), 0.33
Slightly satisfied	8 (42.11)	8 (42.11)	3 (15.79)		1.41 (0.66–2.98), 0.36
Dissatisfied	36 (50.0)	21 (29.17)	15 (20.83)		1.47 (0.84–2.59), 0.17
Extremely dissatisfied	10 (50.0)	3 (15.0)	7 (35.0)		1.72 (0.82–3.57), 0.14
	**PHYSICAL ACTIVITY**				
	GOOD (≥120 min)	REGULAR (60 min)	BAD (none)		
SWL Category, *n* (%) ^b^				0.028	
Extremely satisfied	3 (8.57)	9 (25.71)	23 (65.71)		1.0
Satisfied	8 (12.9)	20 (32.26)	34 (54.84)		1.62 (0.58–4.46), 0.35
Slightly satisfied	0 (0)	5 (26.32)	14 (73.68)		2.23 (0.87–5.73), 0.09
Dissatisfied	17 (25.0)	15 (22.06)	36 (52.94)		2.66 (1.05–6.8), 0.039
Extremely dissatisfied	4 (20.0)	10 (50.0)	6 (30.0)		3.87 (1.35–11.1), 0.012

* RR = Relative Risk; ^b^ Data are expressed as n (%), compared using the Chi-square test. A *p*-value < 0.05 was considered statistically significant. Adjusted for age and sex.

**Table 3:** Association between satisfaction with life and quality of life (QoL) with lifestyle parameters.

Parameter Lifestyle	NEGATIVE LIFESTYLE ß (IC95%), *p* Value			
	BAD DIET	SLEEP < 6 H	SCREEN TIME ≥ 4 H	PA None
SWL	−0.18 (−0.28 to −0.09), <0.001	−0.21 (−0.31 to −0.11), <0.001	−0.10 (−0.18 to −0.01), 0.015	−0.10 (−0.19 to −0.01), 0.027
QoL dimensions				
QoL physical	−0.18 (−0.27 to −0.09), <0.001	−0.21 (−0.30 to −0.11), <0.001	−0.09 (−0.17 to −0.01), 0.019	−0.16 (−0.25 to −0.07), <0.001
QoL psychological	−0.18 (−0.27 to 0.09), <0.001	−0.20 (−0.30 to −0.10), <0.001	−0.07 (−0.15 to 0.001), 0.054	−0.20 (−0.30 to −0.10), <0.001
QoL social	−0.11 (−0.26 to 0.02), 0.096	−0.30 (−0.47 to −0.14), <0.001	0.07 (−0.06 to 0.21), 0.286	−0.25 (−0.41 to −0.09), 0.002
QoL environmental	−0.14 (−0.23 to −0.06), 0.001	−0.13 (−0.21 to −0.04), 0.004	−0.02 (−0.10 to 0.04), 0.455	−0.11 (−0.19 to −0.02), 0.010
QoL raw score	−0.06 (−0.09 to −0.03), <0.001	−0.07 (−0.10 to −0.04), <0.001	−0.02 (−0.04 to 0.006), 0.141	−0.06 (−0.09 to −0.03), <0.001

Adjusted model by age and sex. *p*-value < 0.05 was considered statistically significant. SWL = satisfaction with life, QoL = Quality of Life.

## Data Availability

The original contributions presented in this study are included in the article. Further inquiries can be directed to the corresponding author.
